# Host TLR4 activation by tenascin-C is associated with MMP14-dependent glioma invasion

**DOI:** 10.3389/fonc.2026.1915071

**Published:** 2026-09-08

**Authors:** Hannah Haneke, Ibrahim E. Efe, Omar Dzaye, Helmut Kettenmann, Fatih Yalcin

**Affiliations:** 1 Cellular Neurosciences, Max-Delbrück-Center for Molecular Medicine in the Helmholtz Association, Berlin, Germany; 2 Department of Hematology, Oncology and Tumor Immunology, Charité Universitätsmedizin Berlin, Berlin, Germany; 3 Johns Hopkins University, Russell H. Morgan Department of Radiology and Radiological Science, Baltimore, MD, United States; 4 Shenzhen Institute of Advanced Technology, Chinese Academy of Sciences, Shenzhen, China; 5 Institute of Pathology, Christian-Albrecht University of Kiel, Kiel, Germany; 6 Department of Neurosurgery, University Medical Center Schleswig-Holstein, Kiel, Germany

**Keywords:** glioblastoma, glioma invasion, innate immune activation, MMP14, tenascin-C (TNC), toll-like receptor 4, tumor immune microenvironment, tumor-associated microglia/macrophages (TAM)

## Abstract

Glioblastoma is the most aggressive primary brain tumor in adults and is characterized by diffuse invasion and profound remodeling of the tumor microenvironment. Tumor-associated microglia and macrophages represent the largest non-malignant population in glioblastoma and promote invasive growth through context-dependent inflammatory and matrix-remodeling programs, yet the endogenous microenvironmental cues that drive their pro-invasive activation remain incompletely defined. Tenascin-C (TNC) is an extracellular matrix glycoprotein abundantly expressed in glioblastoma and a reported endogenous ligand of Toll-like receptor 4 (TLR4). Here, we investigated whether tumor-derived TNC engages TLR4 signaling in host myeloid cells, consistent with a role to promote glioma invasion through induction of matrix metalloproteinases. To this end, we combined analyses of freshly resected human glioblastoma specimens, public bulk sequencing and CISH datasets, ex vivo GL261-organotypic brain slice cultures from wild-type and TLR4-deficient mice, and primary murine microglia cultures. TLR4 expression in human glioblastoma was enriched in CD11b^+^ tumor-associated myeloid cells and was linked to immunoregulatory rather than pro-inflammatory macrophage signatures. TNC was strongly upregulated in tumor tissue, derived predominantly from non-myeloid CD45^-^ cells, and was spatially enriched in invasive and perivascular regions, where it was juxtaposed to reactive Iba1^+^ microglia displaying an amoeboid morphology. Across independent glioma cohorts, TNC expression correlated with the membrane-anchored matrix-remodeling protease MMP14, particularly within anatomically defined invasive tumor compartments, and combined high TNC and MMP14 expression was associated with shorter overall survival. Functionally, host TLR4 deficiency reduced glioma growth and infiltrative expansion in organotypic brain slices, and in primary microglia, TNC induced a robust upregulation of MMP14 that was markedly attenuated in TLR4-deficient cells, whereas MMP9 regulation was modest and only partially TLR4-dependent. Cell-type-resolved single-nucleus and ligand-receptor analysis (CellChat/LIANA+) in two independent datasets (BrainTIME, GBM-CARE) further supported directional TNC-TLR4 communication from non-myeloid/malignant to myeloid populations. Together, these findings support a model in which tumor-derived TNC contributes to TLR4 activation in host myeloid cells and is associated with a pro-invasive microenvironmental program involving MMP14 induction in GBM. The identified TNC–TLR4–MMP14 signaling axis links extracellular matrix remodeling with innate immune activation, which highlights a potential tumor immune mechanism contributing to glioma invasion.

## 1 Introduction

Glioblastoma (GBM) is the most aggressive primary brain tumor in adults and remains lethal despite maximal resection followed by radiochemotherapy (1, 2). Even with the current standard-of-care treatment, median survival remains poor, with an average life expectancy of 12–18 months after diagnosis, underscoring the need for a better mechanistic understanding of GBM progression (3–5). Its poor outcome is driven less by bulk tumor growth than by diffuse infiltration into surrounding brain parenchyma, which is a defining feature of GBM thereby limiting surgical control and enabling rapid recurrence (6). This invasive phenotype is not solely tumor cell–autonomous but is critically shaped by interactions with non-malignant components of the tumor microenvironment (TME), particularly tumor-associated microglia and macrophages (TAMs) (7, 8).

These myeloid cells comprise up to 30–50% of the tumor mass and accumulate within the tumor core as well as the invasive edge, where they adopt context-dependent phenotypes that promote tumor progression, ECM remodeling and treatment resistance (9–11). The endogenous signals that initiate and spatially organize this reprogramming remain incompletely understood.

Pattern-recognition receptors of the Toll-like receptor (TLR) family have emerged as important mediators of glioma–microglia crosstalk. Previous work, including studies from our group, has shown that glioma-derived endogenous ligands can engage TLR signaling pathways in microglia to promote tumor-supportive functions, including the upregulation of ECM-remodeling proteases (12–16). In particular, TLR2-dependent signaling has been implicated in the induction of microglial matrix metalloproteinases (MMPs) that facilitate glioma invasion (12, 16). By contrast, the contribution of TLR4 signaling to microglial reprogramming in GBM, and the identity of its relevant endogenous ligands in this context, remain less well defined despite the broader importance of TLR4 as a sensor of endogenous damage-associated and exogenous pathogen-associated molecular patterns (17–20).

Tenascin-C (TNC) is a large ECM glycoprotein that is minimally expressed in the adult brain but strongly upregulated in GBM, where it has been associated with invasive growth and adverse clinical outcome (21, 22). Beyond its structural role, TNC can function as an endogenous danger signal: its fibrinogen-like globular (FBG) domain has been shown to activate TLR4 on myeloid cells and modulate innate immune responses in a context-dependent manner (18, 19, 23, 24). TNC has attracted translational interest as a tumor-associated target in GBM, but its functional contribution to microglia-mediated invasion remains insufficiently resolved (25, 26). In particular, it remains unclear whether TNC acts as a functionally relevant TLR4 ligand in tumor-associated microglia and which downstream effector mechanisms link this interaction to glioma invasion.

One potential effector of microglia-mediated invasion is membrane-type 1 matrix metalloproteinase (MT1-MMP/MMP14), a membrane-anchored protease that enables pericellular ECM degradation and invasive tissue remodeling (27, 28). MMP14 is enriched in tumor-associated myeloid cells in glioma and has been linked to invasive growth; however, knowledge of the upstream microenvironmental cues that regulate its expression in microglia within the GBM TME remains limited (9, 16, 29, 30).

In this study, we investigated whether tumor-derived TNC functions as an endogenous ligand that engages TLR4 signaling in host myeloid cells to regulate MMP expression consistent with a role in glioma invasion. Using a combination of human tumor profiling, bioinformatic analyses, ex vivo organotypic brain slice models, and primary microglial cultures, we examined the cellular distribution of TLR4 in GBM, the spatial relationship between TNC and invasive tumor regions, and the role of TLR4 signaling in microglial MMP14 regulation. Together, this work aims to define a mechanistic link between tumor-derived ECM cues and microglial effector programs that support glioblastoma invasion.

## 2 Materials and methods

### 2.1 Human subjects

Human glioma samples were obtained from patients undergoing surgery at the Department of Neurosurgery, University Medical Center Schleswig-Holstein, Campus Kiel. The study was approved by the Ethics Committee of the University of Kiel (approval #D477/18) and conducted in accordance with the Declaration of Helsinki (1964) and its later amendments. Informed consent was obtained from all patients. Freshly resected tumor tissue was stored in Dulbecco’s modified Eagle’s medium (DMEM; Invitrogen, Carlsbad, CA, USA) at 4 °C for less than 24 h prior to processing. Samples were either fixed in 4% paraformaldehyde (PFA) for 24 h and embedded in Tissue-Tec at –20 °C or used for magnetic-activated cell sorting (MACS).

### 2.2 MACS sorting

CD11b^+^ samples were generated using magnetic activated cell sorting (MACS). Following Percoll gradient centrifugation, tumor and control cell pellets were resuspended in PBS, containing 0.5% fetal calf serum (FCS) and 2 mM EDTA and labeled with anti-CD11b microbeads (Miltenyi Biotec). The MACS isolation was carried out according to the manufacturer’s instructions and cells were subsequently used for RNA isolation.

### 2.3 RNA isolation and RT-qPCR

Total RNA was isolated using RNA mini kit (Promega, Madison, WI, USA) according to the manufacturer’s instructions. Quality and yield were determined by NanoDrop 1000 (PeqLabBiotechnologie, Erlangen, Germany). cDNA was synthesized using 100 ng total RNA with SuperScript II reverse transcriptase kit (Prime Script RT reagent Kit, Takara, Art.No RR037B). RT-PCR gene amplification was executed in duplicate using SYBR Green PCR mix (Applied Biosystems, Waltham, MA, USA) in conjunction with the 7500 Fast Real-Time PCR System from Applied Biosystems. The primers used were designed for TLR4 (TLR4 human: sense TGGAAGTTGAACGAATGGAATGTG, antisense ACCAGAACTGCTACAACAGATACT) Tenascin-C (TNC; murine: sense GTTTGGAGACCGCAGAGAAGAA, antisense TGTCCCCATATCTGCCCATCA; human: sense ATGTCCTCCTGACAGCCGAGAA, antisense AGTCACGGTGAGGTTTTCCAGC) and TATA-Box Binding Protein (TBP; murine: sense 5´-AAGGGAGAATCATGGACCAG-3´, antisense 5´-CCGTAAGGCATCATTGGACT-3´; human: sense 5´-AGCGCAAGGGTTTCTGGTTT-3´, antisense 5´-CTGAATAGGCTGTGGGGTCA -3´), MMP9 (sense 5′-CATTCGCGTGGATAAGGAGT-3′, antisense 5′-ACCTGGTTCACCTCATGGTC-3′), MMP14 (sense 5′-CATCACTGCCCATGAATGAC-3′, antisense 5′-GTGCCCTATGCCTACATCCG-3′). Data analysis was performed using the 2-ΔΔCT method, and normalization was done relative to ß-actin. Results were expressed as a fold change, normalized to the control group unless otherwise stated.

### 2.4 Animal subjects

The study utilized animals that were treated in compliance with the German Animal Protection Law and received approval from the Regional Office for Health and Social Services in Berlin, Germany (Landesamt für Gesundheit und Soziales, Permit Numbers: X9005/18, T0014/08, X9023/12). All efforts were made to minimize pain and suffering. Adult mice were euthanized using the method of cervical dislocation.

For primary microglial culture preparation and organotypic brain slice preparation wild-type C57BL/6J mice (Charles River Laboratories, Sulzfeld, Germany) as well as TLR4 knockout mice (Tlr4−/−) on a C57BL/6J background were employed. Tlr4−/− mice were produced by Dr. Shizuo Akira from Osaka University, Japan, and sourced from Oriental BioServices Inc., Kyoto, Japan. Neonatal microglia were extracted from all genders, whereas organotypic brain slices were solely derived from males. Supplemental organotypic brain slices were generated using transgenic male mice expressing EGFP under the Csf1r promoter (CSFR1-EGFP, MacGreen). Mice were kept in the animal facility of the Max Delbrueck Center in adherence to the German law for animal protection (LaGeSo G125/17) under a 12-hour dark-light cycle with food and water *ad libitum*.

### 2.5 Organotypic brain slice preparation and tumor inoculation

OBSs were prepared as described previously (16). Briefly, 14-day-old WT or TLR4-KO mice were decapitated, and the brains were sliced into 250 µm sections in a coronal plane using a vibratome (Leica Microsystems, VT1000S, Wetzlar, Germany). Brain slices were collected using a sterile 7 mm diameter plastic pipette and transferred to cell culture inserts with 0.4 µm pores (Becton Dickinson, Franklin Lakes, NJ, USA) that were fitted into the wells of a 6-well plate. Each well was filled with 1 ml of culture medium containing DMEM supplemented with 10% heat-inactivated FCS, 0.2 mM glutamine, 100 U/ml penicillin, and 100 mg/ml streptomycin. After overnight incubation, the medium was replaced with cultivation medium containing the ingredients listed above plus 25% heat-inactivated FCS, 50 mM sodium bicarbonate, 2% glutamine, 25% Hanks balanced salt solution, 1 mg/ml insulin (Invitrogen), 2.46 mg/ml glucose (Braun, Melsungen, Germany), 0.8 mg/ml vitamin C (Sigma-Aldrich), and 5 mM Tris in DMEM (all from Invitrogen).

A total of 5000 mCherry-GL261 cells were injected into the caudate putamen region of each slice at a depth of 150µm in both hemispheres. Careful control of the injection procedure ensured that no cells spilled onto the surface of the slice, which could migrate over the surface rather than invade through the tissue. After 4 days, mCherry-GL261 were washed and fixed with 4% PFA. Tumor volumes were measured by confocal microscopy (LSM710, Carl Zeiss) with z-stack scanning and were reconstructed into 3D models for precise volume evaluation using Imaris.

### 2.6 Immunofluorescent staining and image processing

For murine tumor slices, mice brains were harvested and perfused with phosphate buffered salt solution (PBS) followed by 4% paraformaldehyde (PFA) solution (Sigma-Aldrich). 40 µm free-floating tumor sections were prepared as previously described (12). Slices were washed twice in PBS for 5 min and then blocked with 5% of donkey serum and 0.1% Triton-X (Sigma-Aldrich).

Primary antibodies were applied overnight at 4 °C in the following dilutions: 1:100 for murine and human GPNMB (goat, AF2550, R&D Systems), 1:600 for IBA1 (goat, ab5076, Abcam), 1:600 IBA1 (rabbit, ab178847, Abcam), 1:100 for Ki67 (rabbit, ab16667, Abcam), 1:200 for MHCII (rat, 14-5321-81, Invitrogen), 1:100 for CD8A (rat, 14-0081-82, Invitrogen), 1:200 for GZMB (rabbit, ab4059, Abcam), 1:200 for Foxp3 (rabbit, mAb #12653, Cell Signaling), at 4 °C. As secondary antibody, we used 1:200 anti-rabbit IgG (711-545-152), anti-goat IgG (705-605-147), and anti-rat IgG (712-545-153, all from Dianova, Hamburg, Germany). Nuclei were counterstained with 4,6-diamidino-2-phenylindole (DAPI; Sigma-Aldrich). Images were taken using a confocal microscope (LSM710 & LSM700, Carl Zeiss, Oberkochen, Germany) with 10x, 20x, 40x or 63x oil objectives.

### 2.7 Cell culture

The murine GL261 glioma cell line (sourced from National Cancer Institute, MD, USA) was cultivated in DMEM supplemented with 10% FCS, 2mM glutamine, 100 U/mL penicillin, and 100 mg/ml streptomycin (all procured from Invitrogen). DF-1 cells, purchased from ATCC (Manassas, VA, USA), were cultured at 39 °C following the ATCC’s protocols.

### 2.8 Protein isolation and microglia stimulation

TNC protein was isolated from early postnatal mice through immunoaffinity chromatography, following the methodology described by Faissner and Kruse (31). WT or Tlr4−/− microglial cells were stimulated for a period of 6 hours with TNC (55 nM), LPS (4 ng/ml; procured from Enzo Life Sciences, Lörrach, Germany), or PBS.

### 2.9 Bioinformatic analyses

Publicly available datasets and online platforms were used for all in silico analyses, which were exploratory and hypothesis-generating unless otherwise specified. Gene expression and clinical outcome data of glioblastoma patients were accessed via the GlioVis portal (http://gliovis.bioinfo.cnio.es) (32) using TCGA (HG-U133A; mRNA expression z-scores) (33) and CGGA datasets (34). Expression levels of TLR4, TNC, and MMP14, as well as myeloid, lymphoid, and glioma lineage markers, were retrieved.

Correlation analyses were performed using Pearson’s correlation coefficient. Specifically, correlations were calculated between TLR4 and TAM-associated genes (AIF1/Iba1, ITGAM/CD11b, CD204/MSR1, GPNMB, CD14, PTPRC/CD45), glioma lineage genes (GFAP, OLIG2, EGFR, CDKN2A, CDK4, GLI1), as well as between TNC and MMP14.

Immune cell infiltration and macrophage polarization states were analyzed using the TIMER platform (35), correlating TLR4 expression with M0, M1, and M2 macrophage populations by estimating the relative abundance of macrophage polarization states from bulk transcriptomic data using a reference immune expression profile.

RNA-seq data from sorted cell populations were obtained from the Brain Tumor Immune Microenvironment (BTIME) dataset (36) and accessed via the open-access platform (https://joycelab.shinyapps.io/braintime/), enabling comparison of TLR4 and TNC expression across CD45^-^ cells, microglia, and macrophages in IDH-wildtype gliomas, IDH-mutant gliomas, and brain metastases.

The *in situ* hybridization (ISH) analysis of glioma tissue sections and spatially annotated RNA-Sequencing data were used thorough the Ivy Glioblastoma Atlas Project (Ivy GAP) (https://glioblastoma.alleninstitute.org) (37–39), allowing assessment of TNC and MMP14 expression in defined anatomical compartments (leading edge, infiltrating tumor, microvascular proliferation, cellular tumor, and pseudopalisading necrosis).

Survival analyses were conducted in both TCGA and CGGA datasets. Kaplan–Meier plots were generated for TLR4, TNC, and MMP14 individually as well as for combined co-expression (TNChigh+MMP14high vs. TNClow+MMP14low). Survival differences were evaluated using the log-rank (Mantel–Cox) test.

### 2.10 Single-nucleus transcriptomic analysis of the GBM-CARE cohort

Processed single-nucleus RNA-sequencing data from the GBM Cellular Analysis of Resistance and Evolution (GBM-CARE) cohort were obtained from the Gene Expression Omnibus (GEO; GSE274548) (40). The downloaded Smart-seq2 expression matrix contained 14,401 nuclear expression profiles. One profile containing an invalid expression value was excluded, resulting in 14,400 profiles for the present analysis. Expression values were transformed as log2(TPM/10 + 1).

Because the published fine-grained cell-type annotations could not be unambiguously linked to the identifiers in the downloaded expression matrix, nuclei were assigned to broad cellular compartments using prespecified marker-gene scores. For each nucleus, marker scores were calculated from the mean transformed expression of the corresponding marker genes, and the identity associated with the highest score was assigned. Marker sets comprised EGFR, PDGFRA, OLIG1, OLIG2, and SOX2 for malignant-like cells; GFAP and AQP4 for astrocyte-lineage cells; CD163, MRC1, and F13A1 for monocyte-derived macrophages (MDMs); P2RY12, CX3CR1, and TMEM119 for microglia; MOG, MBP, and PLP1 for oligodendrocytes; RBFOX3 and SYT1 for neurons; PECAM1 and VWF for endothelial cells; and CD3D and CD3E for lymphocytes. Because the distinction between malignant cells and nonmalignant astrocytes was based on transcriptional markers rather than copy-number alterations, these populations are referred to as “malignant-like” and “astrocyte-lineage,” respectively.

Uniform manifold approximation and projection (UMAP) was performed using Scanpy on the 2,000 most variable genes after expression transformation (41). Cell-type-resolved expression of TNC and TLR4 was visualized using UMAP feature plots and dot plots. In the dot plots, dot size represents the proportion of nuclei with nonzero expression of the respective gene, whereas color represents the mean log2(TPM/10 + 1)-transformed expression within each cellular population. These analyses were descriptive and were used to assess the cellular distribution of TNC and TLR4; the corresponding UMAP and expression dot plots are presented in the **Supplementary Data**.

As an additional cross-platform assessment of TNC expression, processed 10x Genomics snRNA-seq data from the GBM-CARE cohort (GSE274546) were analyzed using the cell-type annotations provided by the original authors. This analysis comprised 108,944 nuclei from 51 samples and was restricted to TNC because no nonzero TLR4 values were present in the processed 10x expression matrices available for this analysis. No technical mechanism was assumed to account for this observation.

### 2.11 Ligand–receptor interaction analysis

Candidate communication between TNC-expressing non-myeloid source populations and TLR4-expressing myeloid populations was assessed using the CellChat scoring method implemented in LIANA+ (liana-py v1.8.1) (42, 43). Analyses were performed separately using FACS/MACS-sorted bulk RNA-sequencing profiles from the BrainTIME dataset (36) and the processed Smart-seq2 single-nucleus expression matrix from the GBM-CARE cohort (GSE274548) (40). For the BrainTIME analysis, Tumor_CD45neg samples were defined as the source population and MDM and microglia as target populations. Because CellChat was developed primarily for single-cell expression data, its application to sorted bulk profiles was considered exploratory.

Processed Smart-seq2 expression data from the GBM-CARE cohort were obtained from GSE274548 (40). The deposited TPM matrix comprised 23,686 genes and 14,401 nuclear expression profiles derived from 54 tumor specimens from 26 patients. One profile (SMMG001B_p2_B12.1) contained exclusively missing expression values and was excluded, leaving 14,400 evaluable profiles. Expression values were transformed as log2(TPM/10 + 1). The number of profiles refers to evaluable profiles in the deposited GEO matrix and not to the 10,062 quality-controlled and classified nuclei reported in the original publication. Because transferable QC and cell-type annotations were not included in the downloaded files, broad cellular populations were assigned independently using prespecified marker-gene scores. Markers included EGFR, PDGFRA, OLIG1, OLIG2, and SOX2 for malignant-like cells; GFAP and AQP4 for astrocyte-lineage cells; CD163, MRC1, and F13A1 for MDM; and P2RY12, CX3CR1, and TMEM119 for microglia.

Candidate interactions were obtained from a prespecified LIANA+/OmniPath-based interaction resource, supplemented with TNC–TLR4 on the basis of experimental evidence identifying TNC as an endogenous TLR4 activator (24, 44), that seems to be relevant in Glioblastoma multiforme as well (23). CellChat interaction scores were calculated using trimean aggregation, expression-proportion thresholds of 10% for sorted bulk populations and 5% for single-nucleus populations, and 1,000 one-sided permutations with a random seed of 1337. Software-reported permutation values of zero were reported as P ≤ 0.001, reflecting the resolution of 1,000 permutations. CellChat lr_probs values represent dataset-dependent interaction-strength scores rather than calibrated probabilities and were therefore not compared quantitatively between datasets.

### 2.12 Data analysis and statistics

All statistical analyses were performed with GraphPad Prism 7 (GraphPad Software, San Diego, USA). For two-group comparisons, unpaired two-tailed Student’s t-tests (with Welch’s correction when appropriate) or Mann–Whitney U tests were applied. For paired datasets, paired t-tests with Bonferroni correction were used. For experiments with multiple groups, one-way or two-way ANOVA followed by Tukey’s or Bonferroni’s *post hoc* tests were performed.

For correlation analyses, Pearson’s correlation coefficients were calculated. Survival curves were analyzed using Kaplan–Meier methodology with log-rank testing. For progression data, linear regression analyses were used. Tumor volume and surface area were reconstructed using Imaris (v9.3.1) and QuPath (v0.5.1).

Ligand-receptor bubble plots were generated in RStudio using custom R scripts. Data were imported using the readxl package and visualized with ggplot2, with scaling and svglite for vector-graphic export.

All data are presented as mean ± standard deviation (SD). Statistical significance thresholds were defined as *p < 0.05, **p < 0.01, ***p < 0.001, ****p < 0.0001. Details of statistical tests and sample sizes (n) are indicated in the respective Figure Legends.

## 3 Results

### 3.1 TLR4 is enriched in tumor-associated myeloid cells in human GBM and associates with an immunosuppressive TAM signature

Given previous reports suggesting that Toll-like receptors can be expressed not only by immune cells but also by tumor cells in GBM, we first sought to define the cellular distribution of TLR4 within the glioblastoma tumor microenvironment (45, 46).

**Figure 1:**
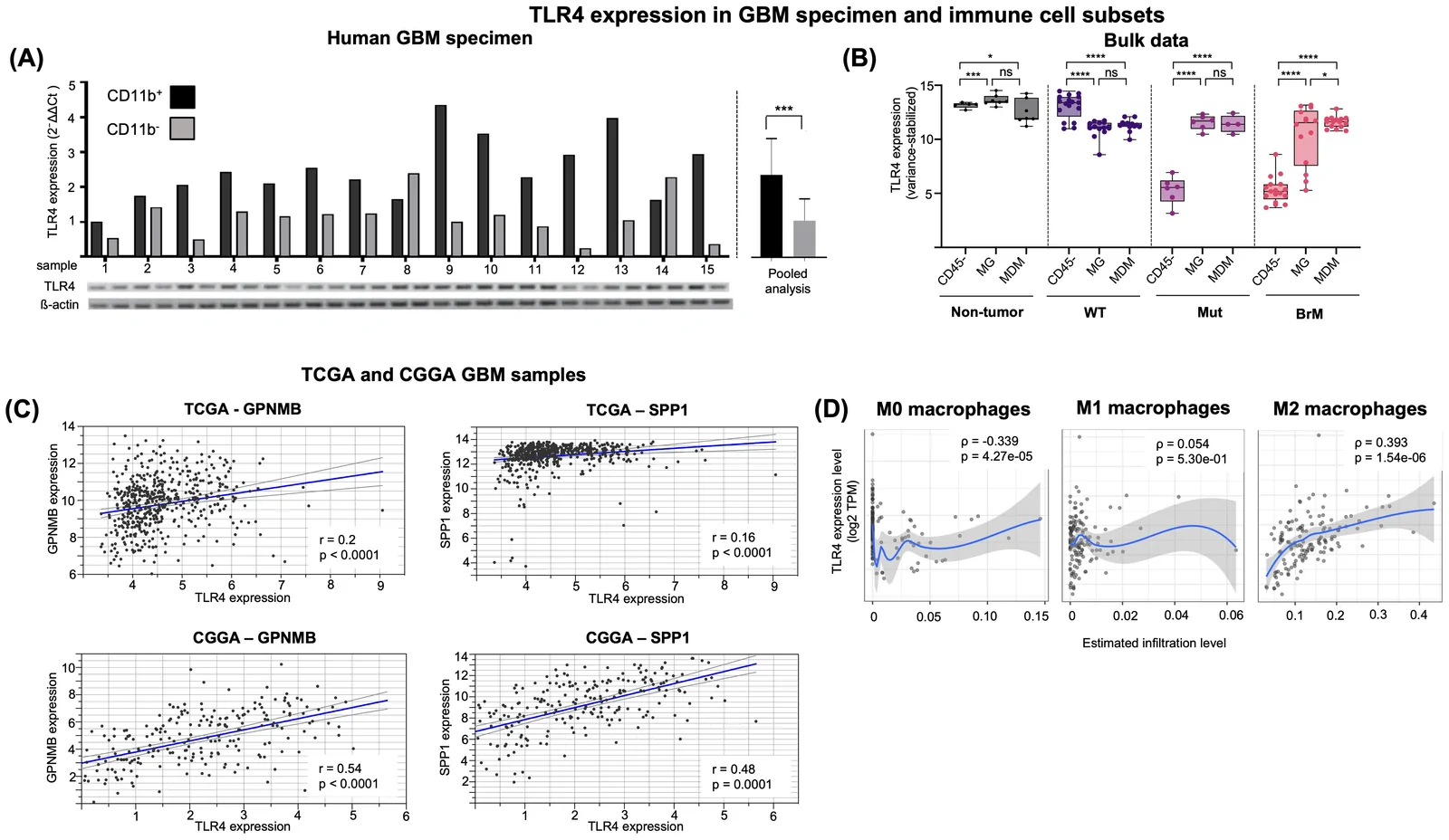
TLR4 is predominantly expressed by TAMs and is associated with immunoregulatory signatures in human glioma. **(A)** TLR4 expression in CD11b^+^ myeloid cells isolated by MACS from freshly resected human GBM specimens and matched CD11b^-^ flow-through fractions (n = 15). TLR4 mRNA was quantified by qPCR (normalized to β-actin) and validated by semi-quantitative PCR. Relative expression was analyzed using the comparative 2^-^ΔΔCt method. An average of all samples is depicted on the right. Statistical analysis was performed using Mann–Whitney U test. **(B)** TLR4 gene expression in CD45^-^ cells, microglia (MG), and macrophages (MPH) from IDH-wildtype glioma, IDH-mutant glioma, brain metastases (BrM), and non-tumor tissue, based on the Brain Tumor Immune Microenvironment dataset (36). TLR4 was enriched in microglia and macrophages compared to CD45^-^ cells across all tumor types. Statistical analysis was performed using two-way ANOVA with Tukey’s multiple comparisons test. Error bars indicate minimum to maximum values. **(C)** In silico Pearson correlation analyses of TLR4 (x-axis) with the TAM-associated markers GPNMB and SPP1, using bulk transcriptomic data from TCGA (HG-U133A dataset, n=528) and CGGA (n=225) glioma samples. TLR4 correlated positively with SPP1 (TCGA: r = 0.16, p = 0.0001; CGGA: r = 0.48, p < 0.0001) and GPNMB (TCGA: r = 0.20, p < 0.0001; CGGA: r = 0.54, p < 0.0001) in both datasets. **(D)** Correlation of TLR4 expression and computationally inferred macrophage polarization states (M0, M1, M2) in the TCGA dataset, estimated by TIMER-based deconvolution of bulk expression profiles (35). TLR4-expression showed a significant negative correlation with M0 macrophages, no significant association with M1 macrophages and a significant positive correlation with M2 macrophages (Spearman’s ρ). *p < 0.05, ***p < 0.001, ****p < 0.0001; ns, not significant.

CD11b^+^ myeloid cells were isolated by magnetic-activated cell sorting (MACS) from freshly resected human GBM specimens (n = 15), and TLR4 transcript levels were quantified by quantitative PCR and validated by semi-quantitative PCR. In 13 of 15 cases, CD11b^+^ cells exhibited higher TLR4 expression than the corresponding CD11b^-^ flow-through fraction. Across all samples, TLR4 expression was more than two-fold enriched in the CD11b^+^ compartment compared with the CD11b^-^ fraction (CD11b^+^: 5.9×10^-^³ vs. CD11b^-^: 2.6×10^-^³ p < 0.001, Mann–Whitney U test; **Figure 1A**).

To validate these findings in an independent dataset, we interrogated the RNA sequencing resource published by Klemm et al. (36). Consistent with our experimental data, TLR4 expression was markedly higher in pre-sorted microglia and macrophages than in CD45^-^ cells across IDH-wildtype and IDH-mutant gliomas, brain metastases, and non-tumor brain tissue (**Figure 1B**). Within glioma samples, TLR4 expression did not show consistent differences between microglia and macrophages, indicating broadly comparable expression across tumor-associated myeloid subsets. In brain metastases, myeloid TLR4 expression showed greater inter-sample variability than in primary glioma (**Figure 1B**), consistent with the heterogeneous tissue and neoplasm of origin and myeloid recruitment programs of metastatic tumors (47). Interestingly, a recent study has identified a breast cancer cell-derived TNC-microglia interaction axis, that has implicated a disease-associated microglia state (48). As brain metastases were included for comparative reference rather than as a primary focus of this study, this observation was not further investigated.

We next assessed, at the tumor level, the relationship between TLR4 and established TAM-associated markers in large glioma datasets via the GlioVis portal (32) using bulk transcriptomic data from TCGA (HG-U133A) (33) and CGGA datasets (34). TLR4 expression correlated positively with *SPP1* (TCGA: r = 0.16, p = 0.0001; CGGA: r = 0.48, p < 0.0001) and *GPNMB* (TCGA: r = 0.20, p < 0.0001; CGGA: r = 0.54, p < 0.0001) (**Figure 1C**), in association to TAM-linked immunoregulatory programs. In line with a myeloid origin, TLR4 also correlated with canonical microglia/macrophage markers (ITGAM/CD11b, AIF1/Iba1, CD14, and PTPRC/CD45), whereas no correlation was observed with markers of non-myeloid lineages (GFAP, OLIG2; **Supplementary Figure 1A**) or selected glioma-associated genes (EGFR, CDKN2A, CDK4, GLI1; **Supplementary Figure 1B**).

Finally, we used TIMER2.0-based immune deconvolution to relate TLR4-expression to inferred M0-, M1- and M2-macrophage infiltration. The method estimates the relative abundance of macrophage polarization states from bulk transcriptomic data using reference immune expression profile (35). TLR4 expression showed a significant negative correlation with inferred M0 macrophages (Spearman’s ρ = -0.339, p < 0.0001), no significant association with pro-inflammatory M1 macrophages (ρ = 0.054, p = 0.53), and a significant positive correlation with immunosuppressive M2 macrophages (ρ = 0.393, p = 1.5 × 10^-6^).

Collectively, these data indicate that TLR4 expression in human GBM is primarily, though not exclusively linked to tumor-associated macrophages and associates with an M2-like, immunosuppressive TAM context rather than pro-inflammatory macrophage activity. At the tumor level, TIMER-based deconvolution of bulk TCGA data (**Figure 1D**) showed an inferred association with an M2-like signature rather than pro-inflammatory macrophage activity; because TIMER estimates immune cell proportions computationally from bulk expression profiles rather than from directly measured cell populations, this finding should be interpreted as a tumor-level association rather than direct evidence of TAM polarization (see Limitations).

Notably, TLR4 expression in the CD11b^-^ subset exceeded that in the matched CD11b^+^ fraction in two specimens (samples #8 and #14, **Figure 1A**). Since tumor cells themselves showed minimal TLR4 expression, a shift in origin toward the tumor compartment is less likely. This deviation can be most plausibly explained by a combination of technical and biological factors: variable tumor cellularity and immune cell composition at the biopsy site, inter-sample differences in tumor purity, or partial cross-contamination during MACS separation, together with genuine inter-patient heterogeneity in TAM composition or GBM subtype, consistent with previous reports of macrophage heterogeneity in glioma (49). Rather than a fundamental exception to the overall enrichment pattern, these two samples illustrate that TLR4 sources can vary between patients, underscoring the need for patient stratification and companion biomarkers in TLR4-targeted therapies.

### 3.2 Host TLR4 promotes glioma growth and invasion in an ex vivo brain slice model

To assess the functional contribution of host-derived TLR4 to glioma progression, we employed an ex vivo organotypic brain slice (OBS) model as previously described (12). mCherry-labeled GL261 glioma cells were inoculated into brain slices derived from wild-type (WT; n animals = 7) or TLR4-deficient (TLR4-KO; n animals = 6) mice. After four days in culture, tumor-bearing slices were fixed and analyzed by confocal microscopy. Tumor volume and surface infiltration area were quantified based on three-dimensional reconstructions of the mCherry fluorescence signal.

**Figure 2:**
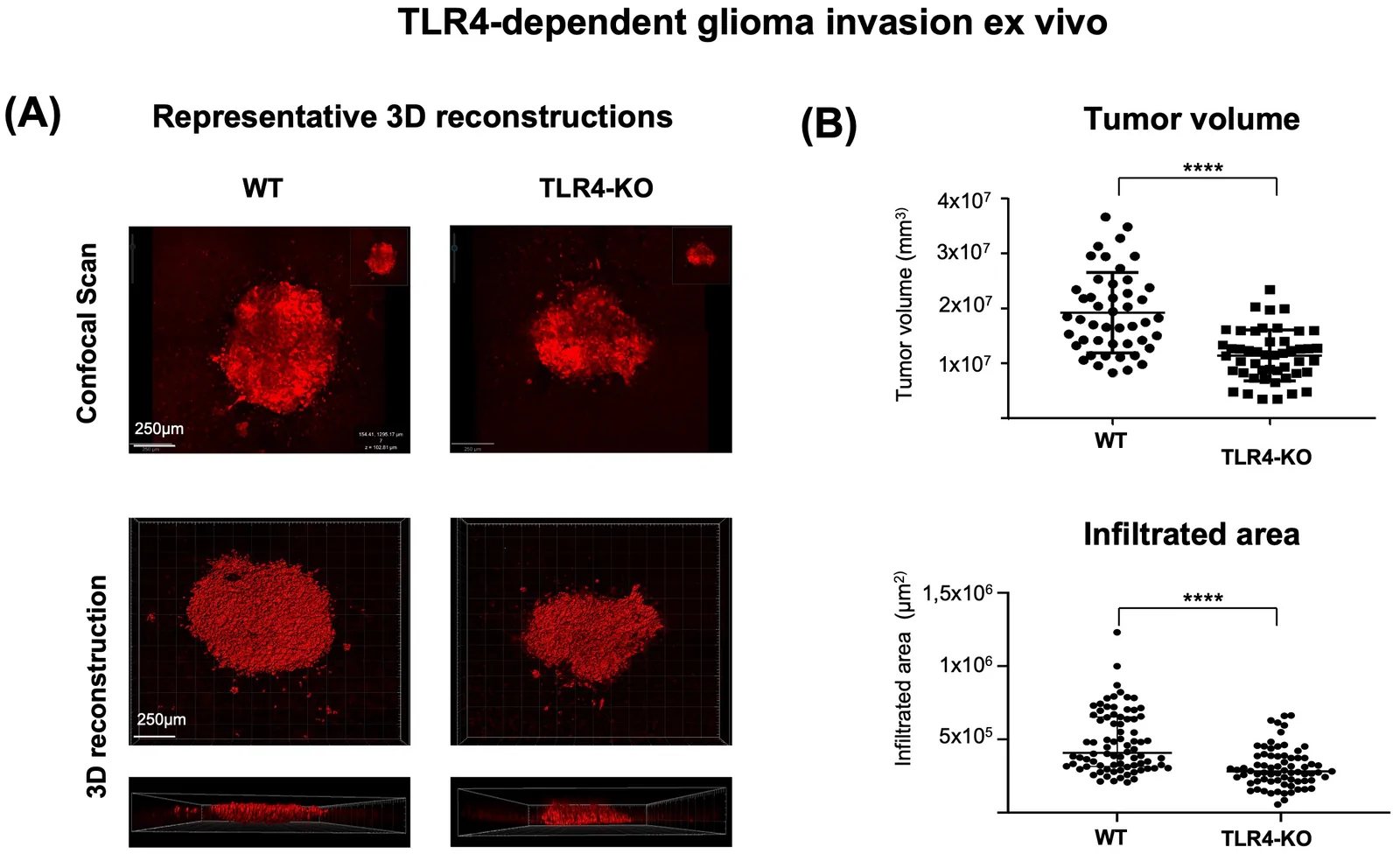
Host TLR4 deficiency reduces glioma expansion and invasion in organotypic brain slices. **(A)** Representative confocal scans and 3D reconstructions of organotypic brain slices (OBS) labeled with mCherry GL261 glioma cells (OBS) from WT (n = 7) and TLR4-KO (n = 6) mice. OBS were obtained from 14–16-day-old C57BL/6 mice and inoculated with 5,000 GL261-mCherry cells. Scans were performed with a Leica SPE confocal microscope using a 10x magnification 5 days after injection. Scale bars: 500 µm. **(B)** Quantification of tumor volume (top, WT n animals = 7, n slices = 47, TLR4-KO n animals = 6, n slices = 48) and tumor surface infiltration area (bottom, WT n animals = 7, n slices = 80, TLR4-KO n animals = 6, n slices = 73) from 3d-reconstructions of mCherry fluorescence signal. Both tumor volume and infiltration area were significantly reduced in TLR4-KO slices compared with WT controls. Each dot represents one tumor-bearing slice; error bars indicate SD. ****p < 0.0001, Welch’s t-test.

Absence of TLR4 in the host tissue was associated with a marked reduction in glioma growth. Tumor volume was reduced by 38.6% in TLR4-KO slices compared with WT controls (p < 0.0001; **Figures 2A, B**, upper panels). In parallel, tumor surface infiltration was significantly decreased in TLR4-KO slices (mean area: 306,894 µm²) relative to WT slices (mean area: 481,457 µm²; mean difference −174,563 ± 28,157 µm²; 95% CI −230,242 to −118,884 µm²). Given unequal variances between groups (F(79,72) = 2.381, p = 0.0002), Welch’s t-test was applied, revealing a highly significant difference (t(136.7) = 6.200, p < 0.0001). The calculated effect size (η² = 0.2195) indicates a substantial contribution of host TLR4 to tumor expansion and invasive behavior (**Figure 2B**, lower panels).

To place these findings in a clinical context, we analyzed the association between TLR4 expression and patient survival at the tumor level, using bulk RNA sequencing data from the TCGA and CGGA glioma cohorts. Patients were stratified into TLR4-high and TLR4-low groups using either median expression or upper-quartile cut-offs. Using the median cut-off, a modest survival difference was observed in the TCGA cohort (median overall survival: 11.7 months for TLR4-high vs. 12.9 months for TLR4-low tumors); however, this association was not maintained when applying the upper-quartile cut-off. Moreover, in primary GBM cases within the TCGA dataset, TLR4 expression was not significantly associated with overall survival using either stratification approach (**Supplementary Figure 2**). These observations indicate that the prognostic impact of TLR4-expression in GBM is limited and dependent on dataset and cut-off selection. Since this survival analysis relies on bulk tumor transcriptomes, it reflects a tumor-level association rather than evidence of a TAM-specific mechanism (see Limitations).

Taken together, these data demonstrate that host-derived TLR4 contributes to glioma growth and invasive expansion in an ex vivo brain tissue context, supporting a pro-tumorigenic role for TLR4 signaling within the glioma microenvironment.

### 3.3 Cellular and spatial expression of tenascin-C in murine and human GBM

Here, we further identified Tenascin-C as a possible TLR4-ligand. Distinct endogenous ligands can elicit divergent TLR4-mediated signaling responses in myeloid cells depending on their molecular and spatial context (50). To identify candidate endogenous TLR4 ligands relevant to glioblastoma, we focused on extracellular matrix–associated molecules with reported immunomodulatory activity in the tumor microenvironment (49). Among these, Tenascin-C (TNC) emerged as a prominent candidate due to its known capacity to engage TLR4 and its established association with tumor invasion (21–23).

**Figure 3:**
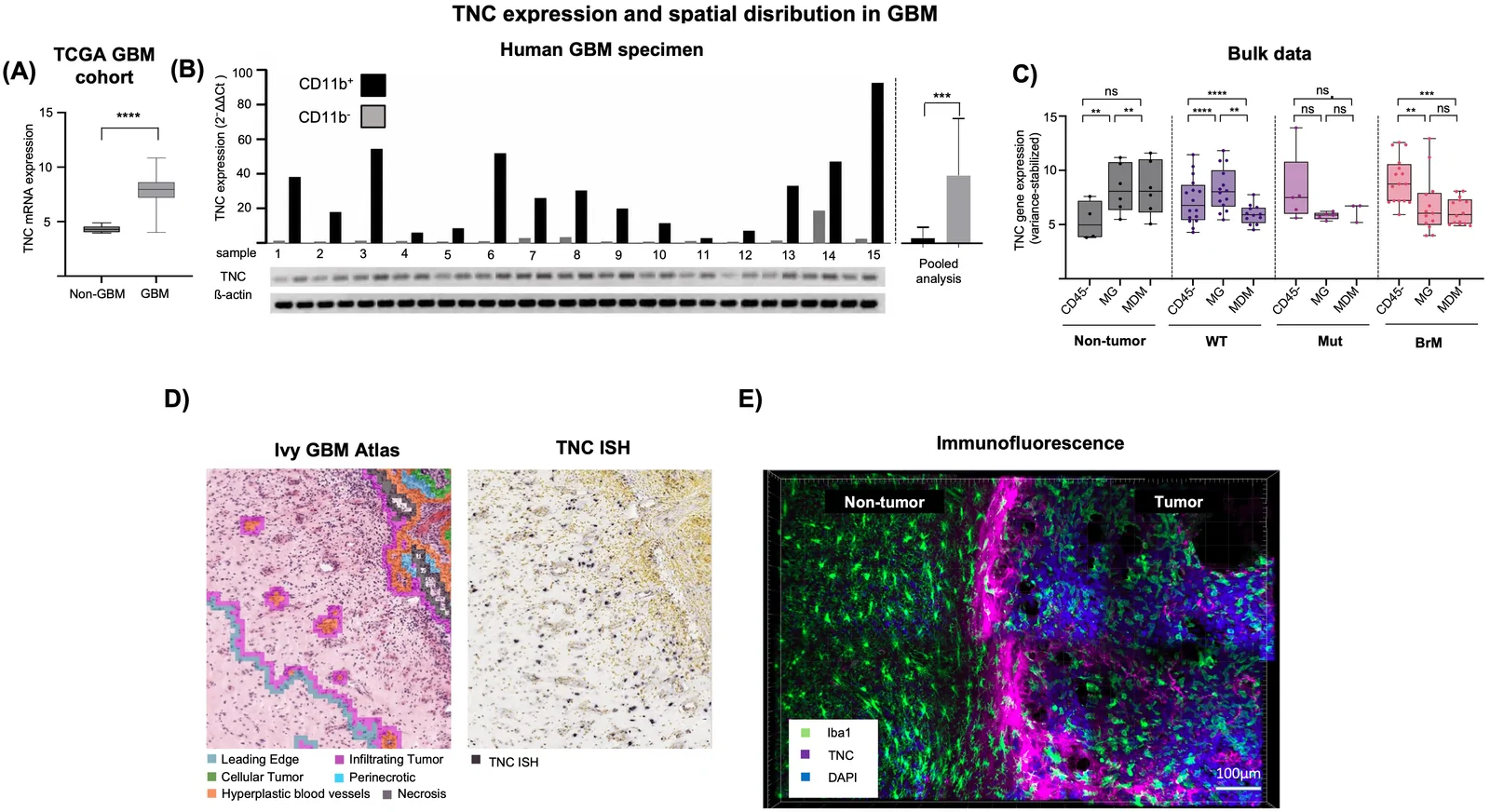
TNC is highly expressed in GBM and accumulates at the invasive tumor front. **(A)** TNC mRNA expression in GBM vs. non-tumor tissue from the TCGA HG-U133A dataset (n = 528 GBM, n = 10 non-tumor). TNC was significantly upregulated in GBM. ****p < 0.0001. **(B)** TNC expression in CD11b^+^ separated by MACS using CD11b vs. CD11b- fractions (flow through) from freshly resected human GBM samples (n = 15) measured by qPCR with ß-actin as the house-keeping gene and further validated by semiquantitative PCR. An average of all samples is depicted on the right. TNC expression was significantly enriched in CD11b^-^ cells. p < 0.0001, paired t-test with Bonferroni correction. **(C)** TNC gene expression in CD45^-^ cells, microglia (MG), macrophages/monocyte-derived macrophages (MDM) across glioma entities (IDHwt, IDHmut) and brain metastases (BrM) from the Brain Tumor Immune Microenvironment dataset (36). TNC expression was consistently higher in CD45^-^ cells and was low to negligible in microglia and macrophages. Statistical analysis: two-way ANOVA with Tukey’s multiple comparisons test. **(D)** Spatial distribution of TNC in the Ivy GBM Atlas (37). Left: Hematoxylin and Eosin (H&E)-stained sections annotated by color for anatomic region. Ivy GBM Atlas histology segmentation showing enrichment of TNC transcripts at the leading edge and infiltrative margins. Right: TNC RNA *in situ* hybridization at the leading edge and infiltrative margins (38). **(E)** Immunofluorescence of GL261 tumors showing co-localization of TNC (purple) with Iba1^+^ microglia (green) and nuclei (DAPI, blue) at the tumor border. 40µm brain slices were prepared from mice inoculated with GL261 cells and scanned with an SPE 20x objective. Scale bar: 100 µm. **p < 0.01, ***p < 0.001, ****p < 0.0001; ns, not significant.

Consistent with this rationale, analysis of bulk transcriptomic data from the TCGA cohort revealed significantly elevated TNC expression in GBM compared with non-tumor brain tissue (mean expression: 7.87 vs. 4.31; p < 0.0001; **Figure 3A**), indicating that TNC is broadly enriched in GBM.

To investigate the cellular source of TNC in GBM, we next analyzed freshly resected human tumors. qPCR analysis of CD11b^+^ and CD11b^-^ fractions from human GBM specimens (n = 15) showed that TNC expression was predominantly confined to the CD11b^-^ compartment, with more than 30-fold higher expression than in CD11b^+^ myeloid cells (p < 0.0001; **Figure 3B**). This finding indicates that TNC is produced by non-myeloid cells within the tumor. This cellular distribution was independently confirmed using the Brain Tumor Immune Microenvironment (BTIME) RNA sequencing dataset, in which TNC expression was consistently enriched in CD45^-^ cells, with negligible expression in microglia or macrophages across glioma subtypes (**Figure 3C**). Together these data identify the neoplastic compartment as the principal cellular source of TNC.

We next examined the spatial distribution of TNC within the glioblastoma microenvironment. The spatial distribution of TNC has been previously reported in cancer stem cells and the vascular and perivascular niche (22, 51). Analysis of spatially annotated histopathology slides from the Ivy Glioblastoma Atlas Project (37, 38) revealed pronounced enrichment of overlayed by RNA *in situ* hybridization of TNC expression at the leading edge and infiltrative tumor regions, in addition to previously described expression within the tumor core and perivascular niches (**Figure 3D**). In the murine GL261 glioma model, immunofluorescence staining confirmed localization of TNC at tumor borders and perivascular regions, where it was closely juxtaposed to Iba1^+^ microglia displaying an amoeboid morphology (**Figure 3E**).

Collectively, these data show that Tenascin-C is highly expressed in GBM, arises predominantly from CD45^-^ tumor-associated cells, and is spatially enriched in infiltrative and perivascular tumor regions, where it is spatially positioned to interact with tumor-associated microglia.

### 3.4 Cell-type-resolved expression analysis supports predicted TNC–TLR4 communication between non-myeloid and myeloid compartments

To independently extend our findings on the cellular distribution of TNC and TLR4, we analyzed processed single-nucleus transcriptomic data from the GBM-CARE cohort (40). Cell identities were very broadly assigned in the Smart-seq2 dataset using predefined marker-gene scores and visualized by uniform manifold approximation and projection (UMAP) (**Supplementary Figure 3A**). This analysis identified malignant-like and astrocyte-lineage populations as well as microglia, monocyte-derived macrophages (MDM), and other non-malignant cell types.

Cell-type-resolved expression analysis confirmed a complementary, although not mutually exclusive, distribution of TNC and TLR4 (**Supplementary Figures 3A, B**). TNC expression was most prevalent in the astrocyte-lineage and malignant-like populations, with detectable expression in 66.6% and 49.9% of cells, respectively. By contrast, TLR4 was most frequently detected in MDM and microglia (29.3% and 27.7%, respectively), compared with 9.6% of malignant-like cells. Together, these data independently support the preferential association of TNC with malignant/astrocyte-lineage populations and of TLR4 with tumor-associated myeloid populations.

**Figure 4:**
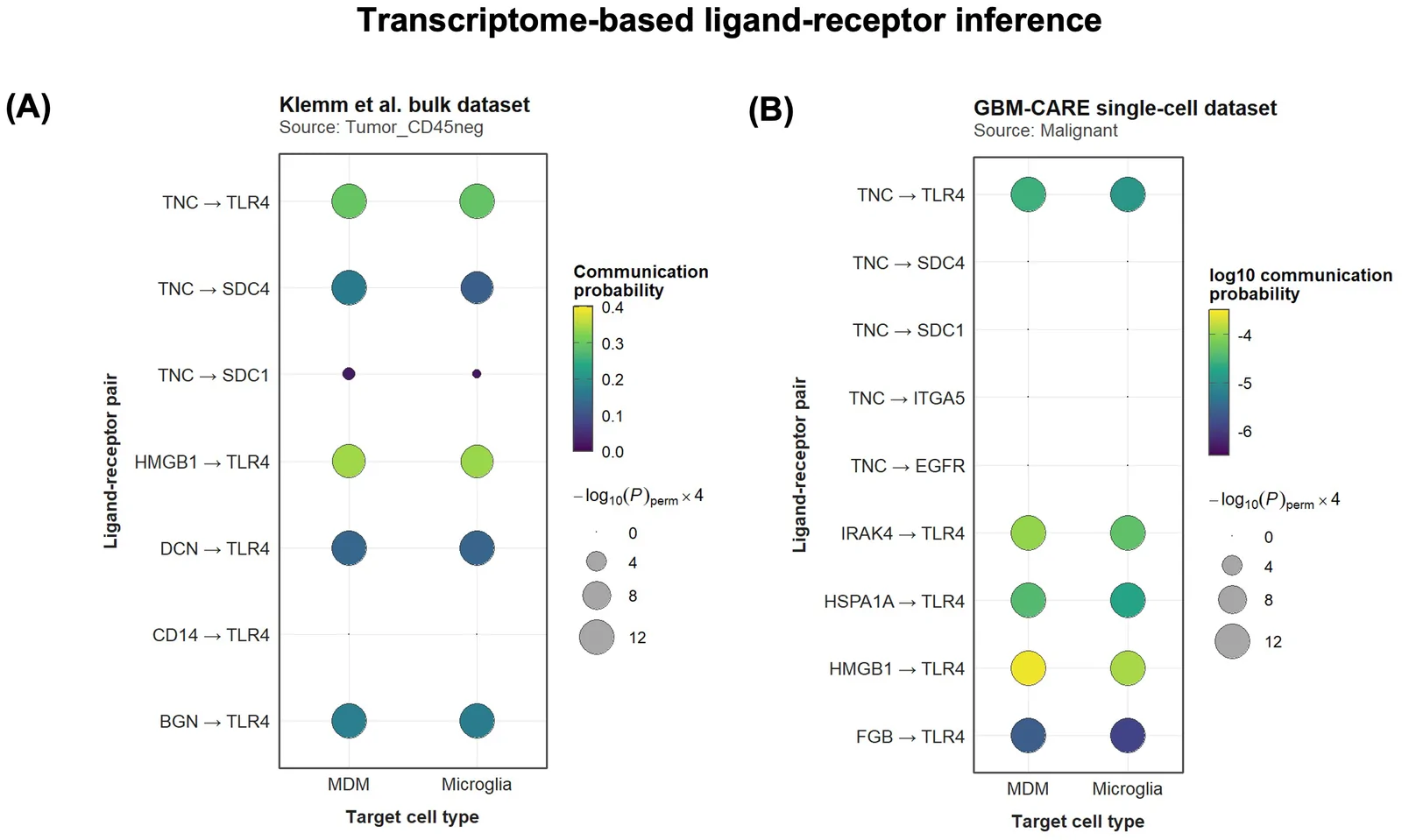
Transcriptome-based ligand–receptor inference supports predicted communication between TNC-expressing malignant cells and TLR4-expressing myeloid cells. CellChat-based ligand–receptor interaction scores were calculated through LIANA+ in the BrainTIME sorted bulk RNA-sequencing dataset and the GBM-CARE Smart-seq2 single-nucleus dataset. **(A)** Predicted interactions from the BrainTIME Tumor_CD45neg compartment toward MDM and microglia. Neutrophils were excluded from the visualization. **(B)** Predicted interactions from the GBM-CARE malignant-like source population toward MDM and microglia. Bubble color represents the CellChat interaction-strength score (lr_probs) on a linear scale in A and log10-transformed lr_probs in **(B)** Bubble area represents −log10(permutation P) × 4 and was capped at 12 for P ≤ 0.001. Because CellChat scores depend on dataset-specific expression distributions and preprocessing, their absolute values and color scales should not be compared quantitatively between datasets.

Having established the cell-type-resolved distribution of TNC and TLR4, we next examined whether the expression pattern was compatible with communication between TNC-expressing malignant cells and TLR4-expressing myeloid cells. CellChat-based ligand–receptor inference was performed through LIANA+ using GBM-CARE Smart-seq2 data and, as an independent comparison, sorted bulk RNA-sequencing profiles from the BrainTIME dataset (36) (**Figure 4**).

In the BrainTIME dataset, TNC–TLR4 communication was predicted from the Tumor_CD45neg compartment toward both MDM and microglia (lr_probs = 0.296 and 0.293, respectively; permutation P ≤ 0.001 for both). HMGB1–TLR4 produced slightly higher interaction scores, while BGN–TLR4, DCN–TLR4, and TNC–SDC4 were also supported by permutation testing.

In the GBM-CARE dataset, TNC–TLR4 communication was predicted from the malignant-like source population toward both MDM and microglia (lr_probs = 2.52 × 10^-5^ and 1.13 × 10^-5^, respectively; permutation P ≤ 0.001 for both). In contrast, the other examined TNC-receptor pairs, including TNC–SDC1, TNC–SDC4, TNC–ITGA5, and TNC–EGFR, were not supported by permutation testing in this dataset. HMGB1–TLR4 was also strongly supported, indicating that the analysis did not identify TNC as the only potential endogenous TLR4-associated signal.

Together, the cell-type-resolved expression analysis and ligand–receptor inference support a model in which TNC-expressing malignant cells may communicate with TLR4-expressing tumor-associated myeloid cells. These transcript-based analyses establish the biological plausibility of the proposed interaction but do not demonstrate spatial contact, physical ligand binding, or receptor activation *in situ*. They therefore provide a rationale for the subsequent functional experiments examining TNC-induced responses in wild-type and TLR4-deficient microglia.

### 3.5 TNC and MMP14 define a spatially pro-invasive axis in GBM

Based on our observation that TNC is enriched at the invasive front of GBM and spatially co-localizes with reactive microglia, we hypothesized that TNC may be linked to microglial effector molecules involved in glioma invasion, building on prior *in vivo* evidence that TNC is enriched at the invasive tumor border and causally promotes invasiveness, though the downstream mechanism linking TNC to invasion in that model was not yet established yet (22).

**Figure 5:**
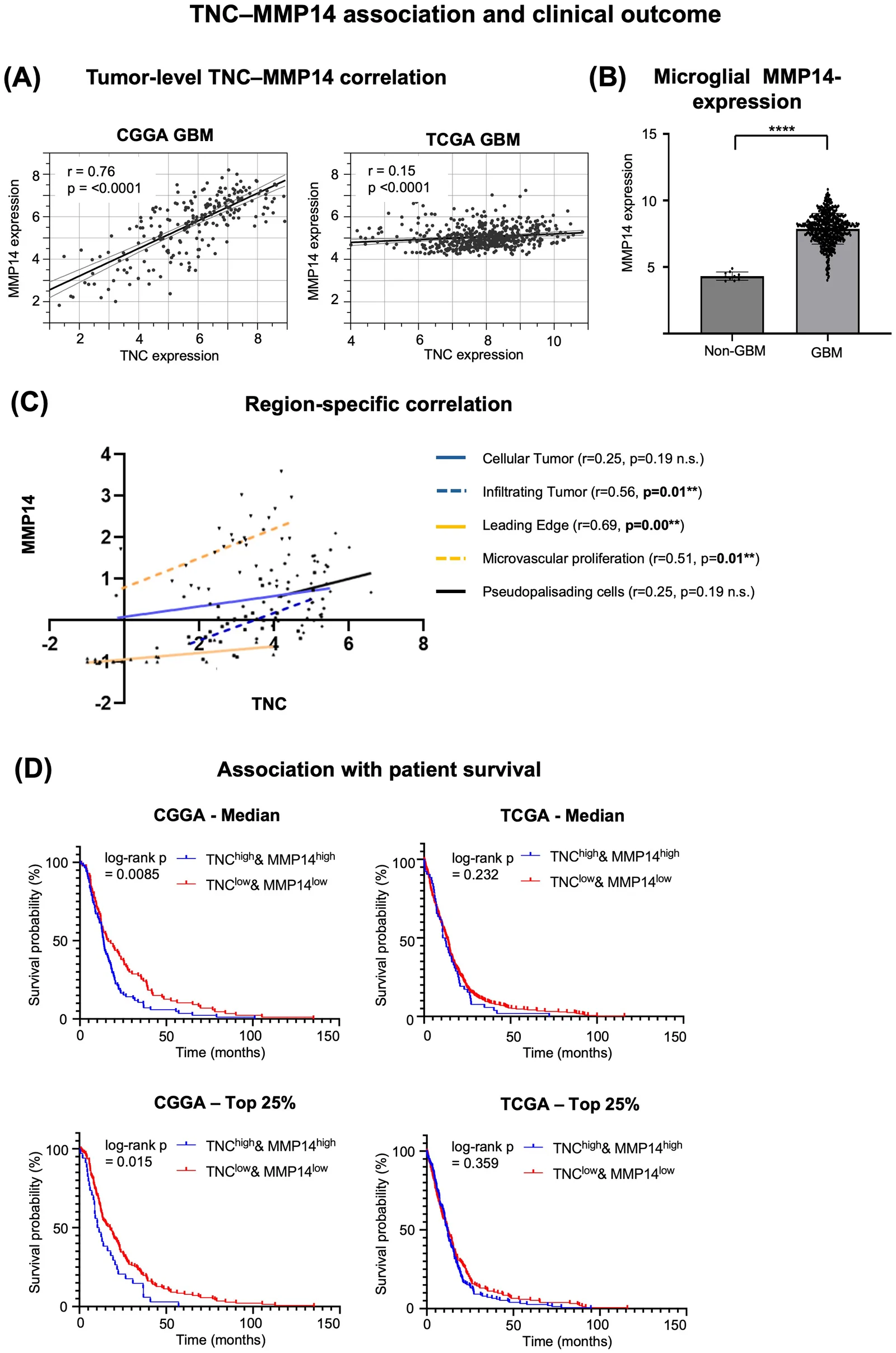
TNC and MMP14 define a spatially enriched pro-invasive signature in GBM. **(A)** Correlation analyses of TNC and MMP14 expression in GBM, using bulk transcriptomic data from the CGGA and TCGA (bottom) datasets. Both datasets revealed a positive correlation between TNC and MMP14 expression (CGGA: r = 0.76, p < 0.0001; TCGA: r = 0.15, p < 0.0001). **(B)** MMP14 expression in tumor-associated microglia/macrophages (TAMs) versus microglia/macrophages from normal brain as reported by (9). MMP14 expression was significantly upregulated in TAMs compared with immune cells from normal brain as quantified by log_2_ fold change (tumor mean = 12.05 vs. normal mean = 10.52; ****p < 0.0001). **(C)** Spatially-resolved tumor-level correlation analysis of TNC and MMP14 expression within anatomically defined GBM regions, that comprise a mix of tumor, stromal and immune cells. The bulk, region-dissected data is derived from the Ivy Glioblastoma Atlas Project (37, 39). The strongest correlations were observed at the invasive leading edge (r = 0.69, p < 0.01), infiltrating tumor (r = 0.56, p = 0.01), and microvascular proliferation zones (r = 0.51, p < 0.01), whereas no significant correlation was observed in the cellular tumor core or pseudopalisading necrosis regions. **(D)** Kaplan-Meier survival analyses, based on bulk TCGA and CGGA transcriptomic data of GBM patients related to co-expression of TNC and MMP14 in the CGGA data set (left) and TCGA data set (right). Patients were grouped into TNChigh+MMP14high versus TNClow+MMP14low expression groups using either median or upper-/lower-quartile expression cut-offs. In the CGGA cohort, high co-expression was associated with shorter survival both at the median cutoff (13.6 vs. 16.4 months; log-rank p < 0.01) and at the top quartile cutoff (10.9 vs. 17.8 months; p < 0.05). In the TCGA dataset, the same trend was observed but did not reach statistical significance.

At the tumor level, in silico analyses of bulk transcriptomic data in the CGGA dataset identified MMP14 as one of the top genes most strongly correlated with TNC expression (r = 0.76; p < 0.0001). The correlation between TNC and MMP14 was validated in the TCGA dataset, where a weaker but significant positive correlation was observed (r = 0.15, p < 0.0001; **Figure 5A**), which likely reflects cohort and platform-specific differences, whereas the concordant direction across both datasets generally strengthens confidence in the association.

To relate this association at the tumor level to a plausible cellular effector mechanism, we next examined a published transcriptomic dataset of tumor-associated microglia. In this dataset, MMP14 was robustly upregulated in tumor-associated microglia compared with microglia/macrophages derived from non-tumor brain tissue (log_2_FC = 1.89; p < 0.001) (9). Thus, among the genes linked to TNC, MMP14 emerged not only as a prominent transcriptomic correlate, but also as a biologically plausible myeloid effector within the GBM microenvironment.

To determine whether the association between TNC and MMP14 is spatially restricted within the glioblastoma microenvironment, we interrogated anatomically dissected, bulk transcriptomic data from the Ivy Glioblastoma Atlas Project (37, 39). This analysis revealed that the correlation between TNC and MMP14 expression was strongest at the leading edge (r = 0.69; p < 0.01) and within infiltrating tumor regions (r = 0.56; p < 0.01), whereas no significant association was detected in the cellular tumor core or in areas of pseudopalisading necrosis (**Figure 5C**). These findings indicate that coordinated TNC and MMP14 expression is preferentially associated with anatomically defined invasive and perivascular compartments of GBM.

To assess potential clinical relevance, we performed Kaplan–Meier survival analyses using bulk RNA-sequencing data from patient cohorts in the CGGA and TCGA datasets. Patients were stratified based on combined TNC and MMP14 expression into high co-expression and low co-expression groups. In the CGGA cohort, high TNC/MMP14 co-expression was associated with significantly reduced overall survival compared with the low-expression group (median survival: 13.6 vs. 16.4 months; log-rank test, p < 0.01). When comparing the upper and lower expression quartiles, this difference was more pronounced, with a median survival of 10.9 months in the high co-expression group versus 17.8 months in the low co-expression group (p < 0.05). In the TCGA dataset, a similar trend was observed; however, survival differences did not reach statistical significance (median survival: 11.9 vs. 12.2 months; **Figure 5D**).

Collectively, these results identify a spatial association between TNC and MMP14 in invasive GBM compartments and link high TNC/MMP14 co-expression to poorer outcome in the CGGA cohort. This prompted us to experimentally test whether TNC directly induces microglial MMP14 expression in a TLR4-dependent manner.

### 3.6 Tenascin-C induces microglial MMP14 expression in a TLR4-dependent manner

To test whether TNC would upregulate MMP14 in microglia, we isolated and stimulated primary cultured microglia from neonatal wild-type (WT) mice with LPS, an exogenous TLR4-ligand, and TNC. As a readout, we focused on MMP14 and MMP9 expression using RT-qPCR, analyzing fold changes in mouse MMP gene expressions through the comparative 2(-DDCt) method relative to β-actin gene-expression levels. The treatment was repeated on microglia cultured from TLR4-knock out (TLR4-KO) mice to determine if the signaling pathway was TLR4-mediated while PBS stimulation served as a control.

**Figure 6:**
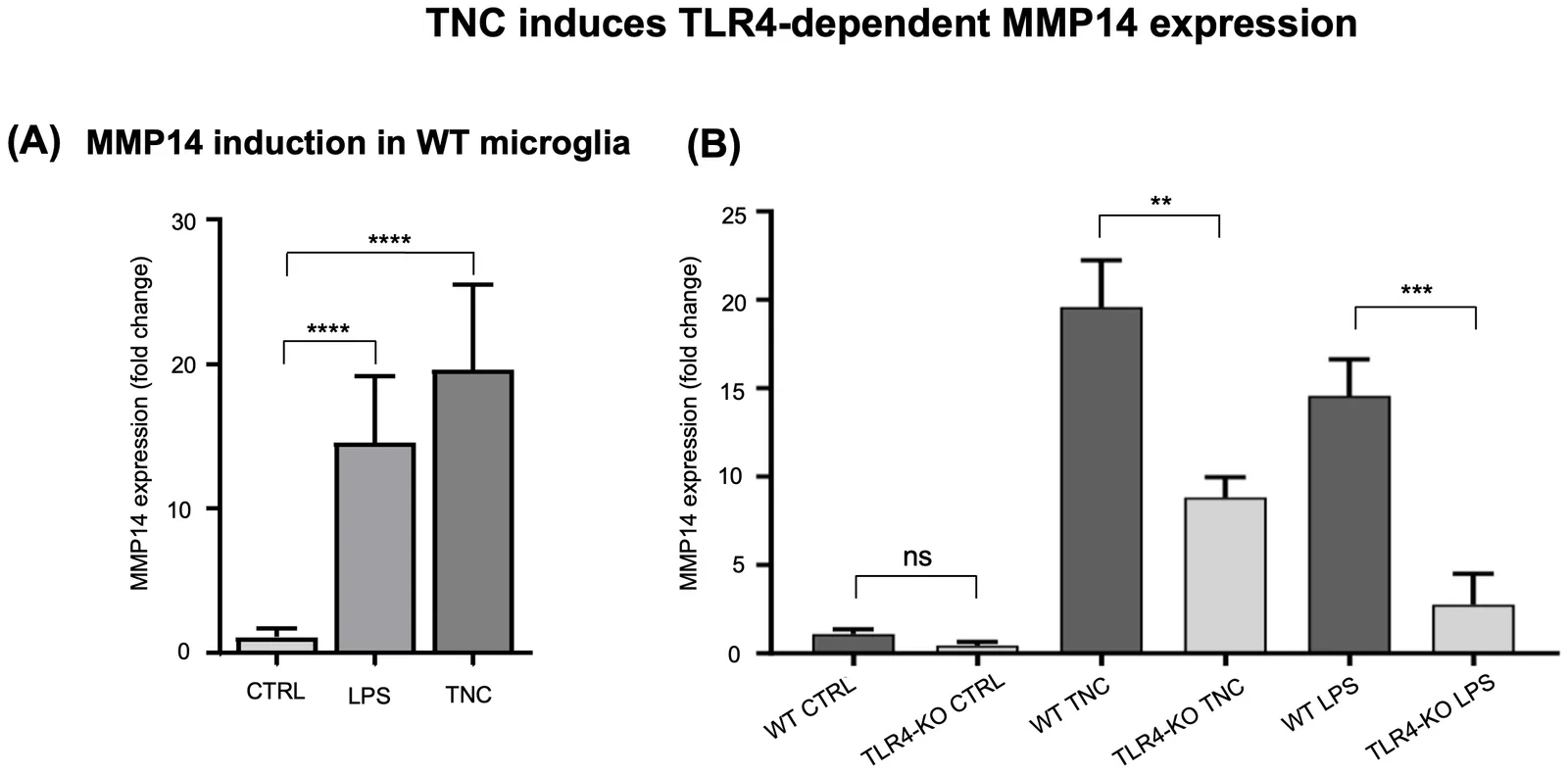
TNC upregulates microglial **(A)** MMP14 induction in WT microglia stimulated with Tenascin-C (TNC) or lipopolysaccharide (LPS); PBS-treated cells served as controls. In WT microglia, both TNC and LPS induced robust MMP14 upregulation, with TNC eliciting the strongest response. **(B)** MMP14 induction in WT vs. TLR4-KO microglia. In TLR4-KO microglia, TNC-induced MMP14 expression was significantly reduced, whereas LPS-induced MMP14 expression was almost completely abrogated, indicating that MMP14 induction by both ligands is TLR4-dependent. MMP14 mRNA expression was quantified by RT-qPCR, normalized to β-actin, and analyzed using the comparative 2^-^ΔΔCt method. Data are shown as fold change relative to PBS-treated WT controls. Data represent n = 5 from at least three independent experiments. Statistical analysis was performed using one-way ANOVA followed by Bonferroni’s multiple-comparisons test. **p < 0.01, ***p < 0.001, ****p < 0.0001; ns, not significant.

TNC stimulation induced a robust upregulation of MMP14 in WT microglia, with an 18.51-fold increase compared to unstimulated controls (p < 0.0001; **Figure 6**). In microglia derived from TLR4-KO-mice, TNC-induced MMP14 expression was reduced by more than 50% compared to TNC-stimulated WT-microglia (10.77-fold vs. 18.51-fold; p < 0.01), indicating that TNC-mediated induction of MMP14 is at least partially TLR4-dependent. Stimulation with LPS, a prototypical exogenous TLR4 agonist, led to a significant increase in MMP14 expression in WT microglia as well (14.65-fold vs. control, p < 0.0001), though the induction was moderately lower compared to TNC. In TLR4-KO microglia, the LPS-induced increase was almost completely abrogated (1.88-fold; p < 0.001 vs. WT LPS), suggesting that both LPS and TNC require TLR4 to mediate MMP14 induction. These findings suggest that endogenous (TNC) and exogenous (LPS) TLR4 ligands elicit quantitatively distinct downstream responses, with TNC eliciting the strongest induction under the conditions tested, which might potentially be explained by ligand-specific signaling kinetics or co-receptor interactions (24, 52).

In contrast, MMP9 expression showed only modest changes following stimulation. TNC treatment caused a small but significant increase in MMP9-expression in WT microglia (1.17-fold, p < 0.05), whereas LPS had no significant effect (0.77-fold, ns; **Supplementary Figure 4**). In TLR4-KO microglia, TNC still induced MMP9 expression (1.52-fold), whereas LPS again had no effect (0.57-fold). These findings suggest that TNC-mediated MMP9 induction occurs at least partially independently of TLR4, whereas LPS does not substantially induce MMP9 under these conditions.

Taken together, these results identify TNC as a potent endogenous inducer of microglial MMP14 expression and show that this response is largely, but not completely, dependent on TLR4. In contrast, TNC-mediated MMP9 upregulation appears modest and may involve additional TLR4-independent pathways. This pattern suggests that ligand-specific TLR4 signaling and complementary TLR4-independent mechanisms may differentially regulate microglial matrix-remodeling effector functions in the glioma microenvironment.

## 4 Discussion

Glioblastoma remains a highly aggressive brain tumor characterized by diffuse invasion, resistance to therapy, and a profoundly immunosuppressive tumor microenvironment. Tumor-associated microglia and macrophages (TAMs) are now recognized as central regulators of glioma progression, yet the endogenous signals that drive their pro-invasive reprogramming remain incompletely understood. In this study, we delineate a mechanistic link between tumor-derived extracellular matrix cues and microglial effector functions, identifying a Tenascin-C–TLR4–MMP14 signaling axis that promotes glioblastoma invasion.

We show that TLR4 expression in human GBM is primarily, but not exclusively, associated with CD11b+ tumor-associated myeloid cells, consistent with earlier reports highlighting immune-restricted TLR4 expression in glioma (53, 54). However, there is a subset of CD11b- samples showing comparable or higher expression (see Limitations). Tumor cells exhibited minimal TLR4 expression, suggesting that modulation of this pathway would primarily affect the immune compartment. Notably, a small subset of samples displayed higher TLR4 expression in non-myeloid fractions, underscoring inter-patient heterogeneity that may reflect differences in TAM composition or molecular subtype, in line with previous observations of macrophage diversity in glioma (49).

Consistent with earlier, partly contradictory studies, TLR4 expression alone did not emerge as a robust prognostic marker across datasets (54, 55), likely reflecting the dual nature of TLR4 signaling, which can promote either inflammatory or immunosuppressive programs depending on context i.e. ligand and microenvironmental cues (18, 19). As these survival analyses are based on bulk TCGA/CGGA transcriptomic data, they do not specifically reflect TLR4 expression within the tumor-associated myeloid compartment and are therefore not directly comparable to the host-microenvironment-specific functional findings described below.

Despite this limited prognostic value, functional experiments in an ex vivo organotypic brain slice model demonstrated that host-derived TLR4 contributes to glioma growth and invasion. Genetic ablation of TLR4 in the brain microenvironment is associated with reduced tumor expansion and infiltrative behavior. These findings are consistent with prior work implicating TLR-dependent microglial pathways in glioma invasion (12), and complementing prior observations by Chicoine et al. (56), who reported similar tumor-size differences without histological changes, and Casili et al. (57), who found contradictory results, in terms of a suppressed tumor growth *in vivo* (U-87 MG xenografts) by TLR4-deficiency (56, 57). Our results support a tumor-promoting role of microglial TLR4 in the OBS model, whereas inconsistencies across models indicate potential model-dependent effects of TLR4, warranting further studies.

Among candidate endogenous TLR4 ligands, Tenascin-C (TNC) emerged as a particularly relevant molecule. TNC is a large extracellular matrix glycoprotein that is strongly upregulated in GBM and has long been associated with invasive growth and poor clinical outcome (51, 58, 59). Previous studies demonstrated that the fibrinogen-like globe domain of TNC can activate TLR4 on myeloid cells, including microglia, in glioma models (22, 23), although the downstream functional consequences of this interaction remained unclear. The TNC–TLR4 interaction is, moreover, mechanistically well characterized at the molecular level: three distinct, cooperatively acting sites within TNC, including the C-terminal fibrinogen-like globe domain, have been mapped as direct TLR4-interaction sites (44). Moreover, Haage et al. (23) could show that in primary murine microglia, TNC regulates multiple TLR4-dependent effector functions, including proinflammatory cytokine and chemokine production, chemotaxis, and phagocytosis, and that reduced TNC expression alters microglial morphology and activation state in glioma-bearing mice *in vivo* (23). Together with our present finding that TNC induces microglial MMP14 expression in a largely TLR4-dependent manner (see below), these observations, alongside the spatially and transcriptionally distinct TNC and TLR4 expression we report here (**Figures 1A–C**, **3B, C**), support a model of TNC–TLR4-mediated intercellular communication between the tumor and myeloid compartments, rather than direct evidence of ligand–receptor engagement *in situ*.

Our data confirm that, in contrast to many other solid tumors where TNC is predominantly stromal, TNC in GBM is largely tumor-cell–derived (60, 61). and is spatially enriched at the invasive front and perivascular niches, where it is closely juxtaposed to activated microglia. This spatial organization provides a biologically plausible framework for TNC–microglia interactions at sites of active invasion, however it presents a spatially-resolved tumor-level association rather than direct evidence for co-expression within a single-cell type, as each anatomical region is comprised by a mix of different cell types such as tumor, stromal and immune cells. A plausible explanation for this pattern is that the cellular tumor core and pseudopalisading necrosis represent a poorly vascularized, hypoxic niche that likely limits the microenvironmental cues required for MMP14 induction (62, 63). Myeloid cells in these regions also differ in terms of ontogeny from those at the invasive margin, where microglia predominate. On the other hand, bone marrow-derived macrophages preferentially localize to perivascular and necrotic areas with a distinct, hypoxia-associated activation profile (64). This regional divergence in vascularization and myeloid identity may explain why coordinated TNC and MMP14 expression is restricted to the more vascularized, myeloid-rich invasive and perivascular regions.

A central finding of this study is that TNC potently induces microglial expression of membrane-type 1 matrix metalloproteinase MMP14 in a largely TLR4-dependent manner. This observation extends previous work identifying other glioma-derived TLR ligands, such as versican and GDNF, as regulators of microglial metalloproteinase expression (14, 16). MMP14 is a membrane-anchored protease that enables pericellular matrix degradation and activation of pro-MMP2, thereby facilitating invasive tissue remodeling (29, 65). Its enrichment in tumor-associated myeloid cells and its established contribution to glioma invasion underscore its functional relevance (9, 30). In contrast, TNC-induced regulation of MMP9 occurred largely independently of TLR4, indicating that distinct signaling pathways govern different metalloproteinase programs within microglia.

The clinical relevance of this pathway is supported by bulk transcriptomic analyses demonstrating co-regulation of TNC and MMP14 in human glioma datasets and their spatial association at the invasive margin in the Ivy Glioblastoma Atlas. Co-expression of these genes was associated with reduced survival in the CGGA cohort, consistent with a role for this axis in aggressive tumor behavior. However, as these correlation and survival analyses derive from bulk tumor transcriptomes, they should be interpreted as tumor-level associations rather than direct evidence of a TAM-mediated mechanism. Stronger support for the proposed cell-type distinction comes from our cell-type-resolved data, in which TLR4 and TNC showed reciprocal myeloid versus non-myeloid expression patterns in MACS-sorted human specimens (**Figures 1A**, **3B**) and the BrainTIME dataset (**Figure 1B**). This expression pattern was independently reproduced in the GBM-CARE Smart-seq2 dataset, in which UMAP and dot-plot analyses demonstrated preferential TNC expression in malignant-like and other non-myeloid populations and enrichment of TLR4 in MDMs and microglia (**Supplementary Figure 3**). In addition, CellChat scoring implemented in LIANA+ identified the prespecified TNC–TLR4 interaction from the Tumor_CD45neg compartment in BrainTIME and from malignant-like cells in GBM-CARE toward both MDMs and microglia (nominal empirical P ≤ 0.001 for both target populations in both datasets; **Figure 4**. Collectively, our findings integrate and extend prior observations on TNC as a pro-invasive extracellular matrix molecule, highlighting its dual role in shaping tumor cell behavior and immune cell function. By linking tumor-derived TNC to microglial TLR4 activation and induction of MMP14, our findings are consistent with a model in which ECM-remodeling and innate immune signaling in host myeloid cells converge to support glioblastoma invasion (see limitations). Given the tumor-restricted expression of TNC and the predominant localization of TLR4 to TAMs, targeting this signaling axis may represent a strategy to selectively modulate the invasive tumor microenvironment.

In conclusion, this study identifies Tenascin-C as a tumor-derived endogenous ligand that engages TLR4 signaling in host myeloid cells, consistent with a contribution to MMP14 induction and glioblastoma invasion. By defining this TNC–TLR4–MMP14 axis, we advance the understanding of glioma–microglia crosstalk and provide a framework for future studies aimed at therapeutically targeting invasion.

### 4.1 Limitations

Several limitations of this study should be acknowledged.

First, while we report robust transcriptional regulation of MMP14 in microglia, protein-level validation and additional functional assays beyond tumor volume and surface infiltration remain necessary to fully establish causality between TLR4–TNC signaling and invasive behavior. Accordingly, mechanistic conclusions should be interpreted with appropriate caution.

Second, we relied on a whole-body TLR4-knockout mouse for the functional experiments rather than a microglia- or myeloid-specific conditional knockout. Consequently, the reduced glioma growth and invasion observed in TLR4-deficient organotypic brain slices reflect a contribution of host TLR4 more broadly and cannot be attributed to microglial TLR4 signaling specifically, as other TLR4-expressing host cell types present in the organotypic brain slices may also contribute to this phenotype.

Third, CD11b was used to isolate tumor-associated myeloid cells by magnetic-activated cell sorting; however, CD11b is not exclusively restricted to TAMs and is also expressed on other myeloid populations, including monocytic and granulocytic myeloid-derived suppressor cells. The CD11b^+^ fraction analyzed here therefore does not represent a pure TAM population, which may contribute to the inter-sample variability observed, including the subset of CD11b^-^ samples with comparable or higher TLR4 expression. Based on large flow-cytometry-based immunoprofiling cohorts of the glioma microenvironment, however, TAMs constitute the dominant fraction of CD11b^+^ cells, with MDSCs and neutrophils representing a comparatively minor proportion (66).

Fourth, the correlation analyses linking TLR4, TNC, and MMP14 expression to clinical outcome (**Figures 1C, D**, **5D**) were derived from bulk transcriptomic datasets such as TCGA and CGGA, which do not allow gene expression to be resolved at the level of individual cell populations within the tumor microenvironment. These correlations and survival associations should therefore be interpreted as tumor-level associations rather than as direct evidence of TAM-specific biology. Although the added GBM-CARE single-nucleus analysis provides complementary support for the cell-type-associated distribution of TNC and TLR4, definitive confirmation of their interaction within human GBM tissue will require spatial transcriptomic or *in situ* co-localization approaches and functional validation in purified or genetically defined TAM populations.

Fifth, the ligand–receptor analyses are based on transcript abundance and prior interaction knowledge and should therefore be considered hypothesis-supporting. Because TNC–TLR4 was included as a prespecified interaction based on existing molecular evidence, these analyses assess whether its expression is compatible with directional communication between the defined source and target populations rather than independently establishing the interaction. Application of CellChat scoring to the sorted bulk BrainTIME profiles was considered exploratory. In the GBM-CARE analysis, broad cellular populations were assigned using marker-gene scores rather than the classification in the original publication (40). Moreover, the permutation results obtained from the pooled GBM-CARE nuclei do not constitute patient-level statistical inference.

Sixth, our analyses identify Tenascin-C as a principal endogenous TLR4 ligand in this context; however, the specific TNC isoforms or proteolytic fragments responsible for microglial activation were not characterized. This is particularly relevant given that TNC contains cryptic functional domains that can be differentially exposed by proteolytic processing and exert divergent biological effects, including pro-tumorigenic (e.g. TNIIIA2) and anti-tumorigenic (e.g. FNIII14) activities (21, 67, 68).

Seventh, survival analyses yielded variable results across TCGA and CGGA cohorts, underscoring the need for additional patient-level validation in independent datasets to clarify the prognostic relevance of the TNC–TLR4–MMP14 axis.

## 5 Conclusion

In summary, this study identifies a glioma–microglia crosstalk mechanism in which tumor-derived Tenascin-C engages TLR4 signaling in host myeloid cells, consistent with a contribution to MT1-MMP/MMP14 induction and invasive tumor growth. By linking tumor-derived extracellular matrix cues to microglial effector functions, our findings support a model in which extracellular matrix remodeling and innate immune signaling in host myeloid cells converge to promote glioblastoma invasion. The delineation of this TNC–TLR4–MMP14 axis highlights a potential entry point for future strategies aimed at modulating invasion-supportive microenvironmental pathways in GBM, warranting further translational investigation.

## Data Availability

The original contributions presented in this study are included in the article/**Supplementary Material**. Publicly available datasets analyzed in this study can be accessed at the indicated repositories (TCGA, CGGA via GlioVis http://gliovis.bioinfo.cnio.es; Ivy Glioblastoma Atlas Project https://glioblastoma.alleninstitute.org; Brain Tumor Immune Microenvironment dataset https://joycelab.shinyapps.io/braintime/; GBM-CARE single-nucleus RNA-sequencing dataset via the Gene Expression Omnibus, accession numbers GSE274546 and GSE274548, Nomura et al., 2025 (40)). Further inquiries can be directed to the corresponding author.

## Supplementary material

Supplementary Figure 1 Correlation of TLR4 expression with lineage-specific and glioblastoma-associated markers in TCGA GBM samples. Pearson correlation analyses of TLR4 expression (x-axis) with immune cell and glioma-related markers (y-axis) in GBM samples from the TCGA HG-U133A dataset (n = 528). **(A)** Myeloid cell markers: Significant positive correlations were observed between TLR4 and canonical TAM/microglia markers, including AIF1/Iba1 (r = 0.35, ****p < 0.0001), ITGAM/CD11b (r = 0.33, ****p < 0.0001), CD204/MSR-1 (r = 0.44, ****p < 0.0001), GPNMB (r = 0.21, ****p < 0.0001), CD14 (r = 0.30, ****p < 0.0001), and PTPRC/CD45 (r = 0.38, ****p < 0.0001). **(B)** Non-myeloid/glioma-associated markers: No relevant correlations were detected between TLR4 and astrocytic or glioma lineage-associated genes, including GFAP (r = 0.06, ns), OLIG2 (r = –0.02, ns), EGFR (r = –0.05, ns), CDKN2A (r = 0.01, ns), CDK4 (r = –0.02, ns), and GLI1 (r = 0.00, ns).

Supplementary Figure 2 Prognostic analysis of TLR4 expression in GBM. Kaplan–Meier survival curves of glioblastoma patients from the TCGA (top) and CGGA (bottom) cohorts stratified into TLR4-high (blue) and TLR4-low (red) groups. Patients were dichotomized based on either the median expression (left panels) or the top 25% expression quartile (right panels) of TLR4 mRNA. Survival was assessed using the log-rank (Mantel–Cox) test. In both datasets, no statistically significant survival differences were detected between TLR4-high and TLR4-low groups (all p > 0.05).

Supplementary Figure 3 Cell-type-resolved expression of TNC and TLR4 in the GBM-CARE single-nucleus dataset. Single-nucleus RNA-sequencing data (Smart-seq2) from the GBM-CARE cohort (Nomura et al., 2025; GSE274548; n = 14,400 evaluable nuclear expression profiles from 54 tumor specimens) were used to assess the cell-type-resolved expression of TNC and TLR4. Broad cellular identities were assigned using prespecified marker-gene scores (malignant-like: EGFR, PDGFRA, OLIG1, OLIG2, SOX2; astrocyte-lineage: GFAP, AQP4; monocyte-derived macrophages (MDM): CD163, MRC1, F13A1; microglia: P2RY12, CX3CR1, TMEM119). **(A)** Uniform manifold approximation and projection (UMAP) of GBM-CARE nuclei, colored by assigned cell-type identity, identifying malignant-like and astrocyte-lineage populations as well as microglia, monocyte-derived macrophages (MDM), and other non-malignant cell types. **(B)** Dot plots showing TNC and TLR4 expression across the identified cell populations. Dot size represents the proportion of cells with detectable expression per population, and color represents mean expression level. TNC expression was most prevalent in the astrocyte-lineage and malignant-like populations (66.6% and 49.9% of cells with detectable expression, respectively), whereas TLR4 was most frequently detected in MDM and microglia (29.3% and 27.7%, respectively), compared with 9.6% of malignant-like cells.

Supplementary Figure 4 TNC induces modest microglial MMP-9 expression partially independent of TLR4 MMP9-expression in cultured primary microglia. WT (left) and TLR4-KO (right) cells were stimulated as described above. In WT microglia, TNC induced a modest but significant increase in MMP9 expression, whereas LPS had no effect. In TLR4-KO microglia, TNC still upregulated MMP9 expression, while LPS stimulation remained ineffective, indicating that TNC-mediated MMP9 induction occurs at least partially independently of TLR4.
